# Excessive adiposity at low BMI levels among women in rural Bangladesh

**DOI:** 10.1017/jns.2015.32

**Published:** 2016-02-17

**Authors:** Saijuddin Shaikh, Jessica Jones-Smith, Kerry Schulze, Hasmot Ali, Parul Christian, Abu Ahmed Shamim, Sucheta Mehra, Alain Labrique, Rolf Klemm, Lee Wu, Mahbubur Rashid, Keith P. West

**Affiliations:** 1 The JiVitA Project of Johns Hopkins University, Godown Road, Paschimpara, Gaibandha, Bangladesh; 2 Center for Human Nutrition, Department of International Health, Bloomberg, School of Public Health, Johns Hopkins University, Baltimore, MD, USA

**Keywords:** Overweight, Obesity, Percentage body fat, BMI, Bangladeshi women, Bioelectrical impedance analysis, Skinfolds, BIA, bioelectrical impedance analysis, ROC, receiver operating characteristic

## Abstract

Asian populations have a higher percentage body fat (%BF) and are at higher risk for CVD and related complications at a given BMI compared with those of European descent. We explored whether %BF was disproportionately elevated in rural Bangladeshi women with low BMI. Height, weight, mid-upper arm circumference, triceps and subscapular skinfolds and bioelectrical impedance analysis (BIA) were measured in 1555 women at 3 months postpartum. %BF was assessed by skinfolds and by BIA. BMI was calculated in adults and BMI *Z*-scores were calculated for females <20 years old. Receiver operating characteristic (ROC) curves found the BMI and BMI *Z*-score cut-offs that optimally classified women as having moderately excessive adipose tissue (defined as >30 % body fat). Linear regressions estimated the association between BMI and BMI *Z*-score (among adolescents) and %BF. Mean BMI was 19·2 (sd 2·2) kg/m^2^, and mean %BF was calculated as 23·7 (sd 4·8) % by skinfolds and 23·3 (sd 4·9) % by BIA. ROC analyses indicated that a BMI value of approximately 21 kg/m^2^ optimised sensitivity (83·6 %) and specificity (84·2 %) for classifying subjects with >30 % body fat according to BIA among adults. This BMI level is substantially lower than the WHO recommended standard cut-off point of BMI ≥ 25 kg/m^2^. The equivalent cut-off among adolescents was a BMI *Z*-score of –0·36, with a sensitivity of 81·3 % and specificity of 80·9 %. These findings suggest that Bangladeshi women exhibit excess adipose tissue at substantially lower BMI compared with non-South Asian populations. This is important for the identification and prevention of obesity-related metabolic diseases.

Overweight and obesity are associated with increased risk for morbidity^(^^1^^,^^2^^)^, disability^(^^3^^)^ and mortality^(^^4^^)^. Even among low-income countries, the prevalence of overweight and obesity has increased dramatically over the past three decades^(^^5^^)^. The South Asian region has the lowest prevalence of overweight (when defined as BMI ≥ 25 kg/m^2^) for both men and women^(^^6^^)^. In particular, Bangladesh exhibits the lowest mean BMI among females. However, even in Bangladesh, average BMI has increased since 1980^(^^5^^,^^6^^)^. Furthermore, although chronic energy deficiency and infectious diseases are highly prevalent, 41 % of the disease burden^(^^7^^)^ and 52 % of the total mortality is due to non-communicable diseases (excluding injury)^(^^8^^)^. Additionally, CVD and diabetes are relatively high for this context and have increased in recent years^(^^9^^–^^12^^)^.

The combination of very low mean BMI yet substantial non-communicable disease burden may signal that standard BMI-based definitions of overweight do not adequately reflect excess adiposity levels in South Asian populations. For example, comparing white European children with Indian children of the same birth size, Indian children have substantially higher levels of subcutaneous adiposity and lower levels of abdominal viscera and lean tissue^(^^13^^)^. Similar results have been reported among South Asian Indian adult females and males^(^^14^^)^. When adiposity was measured by the sum of skinfold measurements, Indian women had a mean estimated body fat of 35·4 % at a BMI of 23·3 kg/m^2^; the corresponding values for men were body fat of 21·3 % at a BMI of 21·4 kg/m^2^. For comparison, white women have been found to reach an average body fat of 35 % at approximately BMI ≥ 30 kg/m^2^.

Data from East and South-East Asian populations (China, Hong Kong, Indonesia, Japan, Singapore, and urban and rural Thailand) compiled by a WHO Expert Committee found higher levels of adiposity and substantial cardiovascular risk at lower BMI for some Asian populations compared with white American and European populations. However, there was significant heterogeneity among Asian populations, and this precluded the establishment of one cut-point for all Asian populations. As an alternative, ‘health action points’ of BMI > 23 and 27·5 kg/m^2^ were established since the risk for CVD was higher among some Asian populations at a BMI lower than the traditional WHO cut-off points^(^^15^^)^.

Notably, the South Asian region, including countries such as Bangladesh, India and Pakistan, was not represented in the analyses by the expert committee due to the lack of direct measures of fatness in regional studies. However, using indirect measures, evidence from India^(^^14^^)^ and from South Asians living in New Zealand^(^^16^^)^ suggests that the level of body fat at a given BMI may be substantially higher for South Asians even compared with other Asian populations. Our goal was to add to this literature by examining how BMI corresponds to body fat percentage among a large sample of rural Bangladeshi women of reproductive age. Furthermore, we improve upon the existing South Asian studies by utilising bioelectrical impedance with a population-specific body fat percentage equation derived from comparison with ^2^H_2_O dilution^(^^17^^)^. Among rural Bangladeshi women, we investigate cut-off levels of BMI for defining overweight that coincide with commonly used percentage body fat categorisations.

## Subjects and methods

This study was comprised of participants in a substudy that was nested within a cluster randomised, double-masked, placebo-controlled trial designed to investigate the impact of weekly vitamin A or β-carotene supplementation on maternal and infant health and survival conducted at the JiVitA Maternal and Child Health and Nutrition Research Project in two rural districts of northwest Bangladesh^(^^18^^)^. Substudy participants (approximately 5 % of all women in the original trial) were selected based on area of residence. In the trial, women were enrolled in early pregnancy and followed, through regular visits, to 3 months postpartum. Among other more intensive assessments conducted among women in the substudy, anthropometric status and bioelectrical impedance analysis (BIA) were measured at 3 months postpartum. Prediction equations from resistance measures obtained from BIA were developed against the ^2^H_2_O dilution method (reference method) for calculating body water, from which estimates of body composition were derived based on assumptions regarding the hydration status of fat-free mass^(^^19^^)^.

Sociodemographic characteristics, such as maternal education, employment, living standard index and parity, were collected using a structured questionnaire, and age was calculated at each visit by subtracting birth date from the date of the visit. All anthropometric measurements were completed in the home by trained and routinely standardised female anthropometrists.

Weight with light clothing was measured on solar-powered SECA digital scales to the nearest 200 g (SECA UNICEF Electronic Scale 890). Standing height was measured to the nearest 0·1 cm using a portable Harpenden Pocket Stadiometer (Cromwell), modified with a spirit level affixed to the cross-bar to position subjects along the Frankfort plane. Skinfold thickness (triceps and subscapular) was measured with Holtain calipers (Holtain Ltd) to the nearest 0·2 mm. Mid-upper arm circumference was measured to the nearest 0·1 cm using a non-stretch insertion-type measuring tape manufactured by JiVitA. Quality control of anthropometry data was monitored by a quality-control team that was extensively trained and standardised. Inter- and intra-observer technical error of measurement was calculated, and found to be lower than reference cut-off points^(^^20^^)^. All measurements (except weight) were taken three times and median values were used for analysis.

Resistance (R) and reactance (Xc) were measured in the home using a single-frequency portable bioelectrical impedance analyser (Quantum II RJL System). Details of the measurement procedure have been previously reported^(^^17^^)^.

Fat mass was calculated using two methods: skinfold method and bioelectrical impedance analysis method. For the skinfold method, first density was calculated using skinfold thickness from the logarithm of total skinfold thickness (triceps + subscapular)^(^^14^^)^ and then percentage body fat was calculated from density using Siri's equation (1956)^(^^21^^)^: 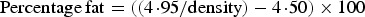


For the BIA method, we calculated percentage fat from BIA as follows. Total body water was calculated using the published equation^(^^19^^)^ which was developed in women at 3 months postpartum in the same community. Fat-free mass (FFM) was derived from total body water by using the hydration factor 0·732^(^^22^^,^^23^^)^. 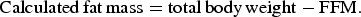


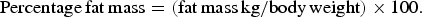


BMI was calculated using the formula weight (kg)/height squared (m^2^). Among females under the age of 20 years (*n* 546), we also calculated age- and sex-specific BMI *Z*-score using the WHO Growth Reference Charts^(^^24^^)^.

This study was conducted according to the Declaration of Helsinki; all study protocols were approved by the Johns Hopkins Bloomberg School of Public Health Institutional Review Board. All included participants provided informed consent.

### Definition of excess body fat

Overweight and obesity are characterised by an excess of body fat or adiposity and this excess adiposity is associated with the increased risk for co-morbidities. Hence, the definition of overweight and obesity should correspond to an amount of body fat that is considered excessive and associated with increased risk for co-morbidities. There is a debate regarding the lower limit of body fat for defining overweight and obesity. However, Deurenberg *et al.*^(^^25^^)^ have produced age- and sex-specific formulas that indicate that, for a 25-year-old white female, a BMI of 25 kg/m^2^ would be expected to correspond to a body fat of 30 %, while a BMI of 30 kg/m^2^ would correspond to approximately 35 % body fat. Too few women in this sample had a body fat percentage that was >35 %; therefore, in this study, >30 % body fat in females (both adolescents and adults) was used as the cut-off for defining overweight or moderately excessive adiposity.

### Statistical analysis

We calculated the frequency distributions of key demographic characteristics of the study population. Pearson's correlation coefficients were calculated between BMI and percentage body fat according to both the skinfold method and the BIA method. Scatterplots were produced to visually assess these relationships.

To assess the BMI and BMI *Z*-score cut-offs that optimally classified women as overweight according to their body fat percentage (≤30 % or >30 %) by the skinfold method and the BIA method, we used receiver operating characteristic (ROC) curve analysis. In ROC analysis, the sensitivity and specificity across a spectrum of cut-offs for BMI/BMI *Z*-score are calculated. We used the ROC analysis to find the BMI and BMI *Z*-score values that resulted in maximising the true positive rate (sensitivity) and minimising the false positive rate (1 – specificity), or equivalently, the value that maximises both sensitivity and specificity. The true positive rate (sensitivity) was plotted against the false positive rate (1 – specificity) across the range of values from the comparison diagnostic test. We also estimated the AUC to assess the overall performance of the BMI and BMI *Z*-score for classifying excessive adiposity. The AUC reflects the probability that the diagnostic test will classify individuals correctly^(^^26^^)^. The ROC analysis was conducted separately for women under and above 20 years, since a substantial proportion of our population was between 14 and 19 years and since age- and sex-specific *Z*-scores, rather than BMI, are typically used to classify weight status at this age.

Finally, a linear regression between BMI/BMI *Z*-score and percentage body fat was performed to assess the magnitude of the association between BMI and body fat. Lowess plots were visually assessed to evaluate if it was reasonable to model these as linear relationships. In addition, squared terms for BMI/BMI *Z*-score were tested and retained in the models if statistically significant.

In supplementary analyses, we used *t* tests to compare the anthropometrics of our study population with previously published results.

Analyses were performed using STATA 11.0 intercooled version (STATA Corporation) statistical software. The significance level was set at <0·05.

## Results

For this substudy, 2668 pregnant women were enrolled during their first trimester. Of them, 1861 women were enrolled at 3 months postpartum period and 1602 women agreed to participate in this study. Of these women, 1555 had completed anthropometries and BIA. Of the total sample, 35·1 % (*n* 546) were adolescents (age <20 years). Demographic characteristics according to age, as well as by BMI and body fat status, are shown in Tables 1 and 2. Almost half of the adult women (46·5 %) had no formal education (Table 1); this was lower among adolescents where 22·6 % had no formal education (Table 2). Approximately 20 % of adult women, but 90 % of adolescents, were primiparous.

**Table 1.** Demographic characteristics of study participants according to age, BMI and body fat status: women aged 20 years and above (Numbers and percentages; mean values and standard deviations)

			BMI (kg/m^2^)						Fat percentage†				Fat percentage‡			
	Overall (*n* 1009)		<18·5 (*n* 374)		18·5–22·9 (*n* 560)		≥23 (*n* 74)		≤30 % (*n* 882)		>30 % (*n* 126)		≤30 % (*n* 898)		>30 % (*n* 110)	
Variables*	*n*	%	*n*	%	*n*	%	*n*	%	*n*	%	*n*	%	*n*	%	*n*	%
Education																
None	468	46·5	197	52·8	255	45·5	16	21·6	438	49·7	30	23·8	435	48·5	33	30·0
Primary	226	22·4	85	22·8	131	23·4	10	13·5	198	22·5	28	22·2	202	22·5	24	21·8
≥Secondary	313	31·1	91	24·4	174	31·1	48	64·9	245	27·8	68	54·0	260	29·0	53	48·2
Employed																
No	541	53·7	196	52·4	310	55·4	35	47·3	474	53·7	67	53·2	488	54·3	53	48·2
Yes	467	46·3	178	47·6	250	44·6	39	52·7	408	46·3	59	46·8	410	45·7	57	51·8
Parity																
0	200	19·8	64	17·1	119	21·3	17	23·0	173	19·6	27	21·4	180	20·0	20	18·2
1	315	31·3	118	31·6	176	31·4	21	28·4	280	31·8	35	27·8	280	31·2	35	31·8
≥2	493	48·9	192	51·3	265	47·3	36	48·6	429	48·6	64	50·8	438	48·8	55	50·0
Breastfed	712	99·6	256	99·6	413	99·5	43	100·0	640	99·5	72	100·0	633	99·5	79	100·0
Did not perform physical labour§	677	69·2	243	67·3	384	70·5	50	69·4	586	68·5	91	74·6	603	69·0	74	71·2
LSI‖																
Lowest	278	27·6	132	35·3	142	25·4	4	5·4	267	30·3	11	8·7	260	29·0	18	16·4
2	248	24·6	106	28·3	132	23·6	10	13·5	233	26·4	15	11·9	237	26·4	11	10·0
3	240	23·8	85	22·7	142	25·4	13	17·6	214	24·3	26	20·6	211	23·5	29	26·4
Highest	242	24·0	51	13·6	144	25·7	47	63·5	168	19·1	74	58·7	190	21·2	52	47·3
Age (years)																
Mean	26		26		26		28		26		28		26		27	
sd	5·5		5·8		5·2		6·2		5·4		5·9		5·6		5·4	
Weight (kg)																
Mean	43·6		38·8		45·0		56·6		42·2		53·0		42·4		53·0	
sd	6·2		3·1		4·3		6·2		4·8		7·0		5·0		7·2	
Height (cm)																
Mean	149·6		149·5		149·5		150·6		149·4		150·7		149·4		151·1	
sd	5·1		4·9		5·4		4·9		5·1		5·1		5·1		5·0	

LSI, living standard index.

* Of the total analytic sample ≥20 years old, missing information on these descriptive characteristics was as follows: education *n* 2; employed *n* 1; parity *n* 1; breastfeeding *n* 296; physical labour *n* 31; LSI *n* 1.

† Fat percentage measured by the skinfold thickness method.

‡ Fat percentage measured by the bioelectrical impedance analysis method.

§ Physical labour was defined as carrying heavy objects or working in the fields or husking grain at least 1 d during the past week of visit.

‖ LSI is expressed as quartiles.

**Table 2.** Demographic characteristics of study participants according to age, BMI and body fat status: women aged under 20 years (Numbers and percentages; mean values and standard deviations)

			BMI *Z*-score						Fat percentage†				Fat percentage‡			
	Overall (*n* 546)		<−1 (*n* 226)		≥−1 and <0 (*n* 267)		≥0 (*n* 53)		≤30 % (*n* 529)		>30 % (*n* 17)		≤30 % (*n* 514)		>30 % (*n* 32)	
Variables*	*n*	%	*n*	%	*n*	%	*n*	%	*n*	%	*n*	%	*n*	%	*n*	%
Education																
None	123	22·6	46	20·4	67	25·2	10	18·9	122	23·1	1	5·9	118	23·0	5	15·6
Primary	123	22·6	54	23·9	57	21·4	12	22·6	119	22·5	4	23·5	113	22·0	10	31·3
≥Secondary	299	54·8	126	55·7	142	53·4	31	58·5	287	54·4	12	70·6	282	55·0	17	53·1
Employed																
No	433	79·5	182	80·5	211	79·3	40	75·5	418	79·2	15	88·2	411	80·1	22	68·8
Yes	112	20·5	44	19·5	55	20·7	13	24·5	110	20·8	2	11·8	102	19·9	10	31·2
Parity																
0	491	90·3	202	89·8	239	89·9	50	94·3	475	90·1	16	94·1	461	90·0	30	93·8
1	52	9·6	22	9·8	27	10·2	3	5·7	51	9·7	1	5·9	50	9·8	2	6·3
≥2	1	0·2	1	0·4	0	0·0	0	0·0	1	0·2	0	0·0	1	0·2	0	0·0
Breastfed	430	99·5	179	99·4	216	99·5	35	100·0	422	99·5	8	100·0	407	99·5	23	100·0
Did not perform physical labour§	401	75·1	172	77·1	188	72·9	41	77·4	387	74·9	14	82·4	376	74·9	25	78·1
LSI‖																
Lowest	109	20·0	49	21·7	52	19·6	8	15·1	108	20·5	1	5·9	109	21·3	0	0·0
2	142	26·1	68	30·1	65	24·4	9	17·0	139	26·3	3	17·7	132	25·7	10	31·3
3	147	27·0	56	24·8	74	27·8	17	32·1	142	26·9	5	29·4	139	27·1	8	25·0
Highest	147	27·0	53	23·4	75	28·2	19	35·8	139	26·3	8	47·1	133	25·9	14	43·7
Age (years)																
Mean	17		17		17		17		17		17		17		17	
sd	1·5		1·4		1·5		1·7		1·5		1·8		1·5		1·6	
Weight (kg)																
Mean	41·4		38·3		42·7		48·5		41·2		48·2		41·0		48·1	
sd	4·8		3·5		3·6		4·9		4·6		7·3		4·4		6·0	
Height (cm)																
Mean	148·4		148·5		148·3		148·2		148·4		148·1		148·3		149·3	
sd	5·4		5·6		5·3		4·5		5·4		5·1		5·3		5·9	

LSI, living standard index.

* Of the total analytic sample <20 years old, missing information on these descriptive characteristics was as follows: education *n* 1; employed *n* 1; parity *n* 2; breastfeeding *n* 114; physical labour *n* 12; LSI *n* 1.

† Fat percentage measured by the skinfold thickness method.

‡ Fat percentage measured by the bioelectrical impedance analysis method.

§ Physical labour was defined as carrying heavy objects or working in the fields or husking grain at least 1 d during the past week of visit.

‖ LSI is expressed as quartiles.

Among adult women, compared with their representation in the total population, women with a secondary education were overrepresented among those with BMI ≥ 23 kg/m^2^ and body fat >30 %, while those with no formal education were overrepresented among those with BMI < 18·5 kg/m^2^ and body fat < 30 %. These differences were far less dramatic among adolescents (Tables 1 and 2). On the other hand, for both adult and adolescent women, those in the highest quartile of the living standard index were overrepresented in the highest BMI and body fat categories.

**Fig. 1.**
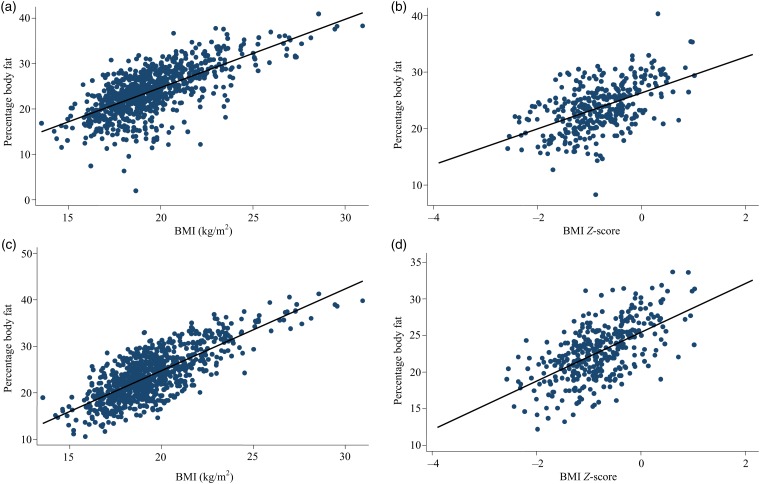
Scatterplot between BMI and body fat percentage by different methods in different age groups: (a) percentage fat by the bioelectrical impedance analysis (BIA) method in women ≥20 years of age; (b) percentage fat by the BIA method in women <20 years of age; (c) percentage fat by the skinfold method in women ≥20 years of age; (d) percentage fat by the skinfold method in women <20 years of age.

The correlation between BMI and body fat percentage as measured by BIA was 0·68 (*P* < 0·001) among adults and 0·54 (*P* < 0·001) among adolescents (using BMI *Z*-score) (Fig. 1(a) and (b)). The correlation between BMI/BMI *Z*-score and body fat percentage estimated by skinfolds was 0·76 (*P* < 0·001) among adults and 0·61 (*P* < 0·001) among adolescents (Fig. 1(c) and (d)). The relationship between BMI and body fat percentage was more variable for body fat measured by BIA than by the skinfold method. The linear regression models indicated that each one unit higher BMI was associated with higher body fat of 1·8 percentage points by the skinfold thickness method and 1·5 percentage points by the BIA method in study women (Table 3). Analogous models for BMI *Z*-scores among participants <20 years old indicate that a one-unit change in BMI *Z*-score was associated with a higher body fat of 3·3 percentage points by the skinfold thickness method and 3·2 percentage points by the BIA method (Table 3). The squared term for BMI *Z*-score was significant in this model and positive indicating increasing magnitudes of association at higher BMI *Z*-score levels. The larger numbers for BMI *Z*-scores stem from the fact that a one-unit increase in *Z*-score is bigger than a one-unit increase in BMI.

**Table 3.** Simple linear regression of percentage body fat on BMI for adults and adolescents

	BMI coefficients	se	*R* ^2^	Constant
Study women ≥20 years				
Percentage fat by skinfold	1·8	0·05	0·59	−10·50
Percentage fat by BIA	1·5	0·05	0·46	−5·43
Study women <20 years				
Percentage fat by skinfold	3·3	0·18	0·38	25·44
Percentage fat by BIA	3·2	0·21	0·29	26·30
BMI *Z*-score squared*	0·41	0·18	0·30	

BIA, bioelectrical impedance analysis.

* BMI or BMI *Z*-score squared was tested in each of the models to allow for curvilinearity. It was only statistically significant in the model of women <20 years where percentage fat was assessed by BIA.

**Fig. 2.**
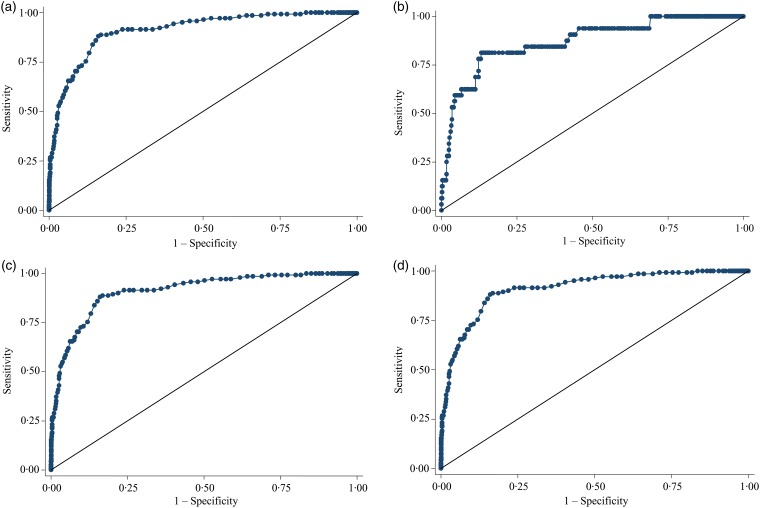
Receiving operating characteristic curve to determine the appropriate cut-off values of BMI (kg/m^2^), while taking percentage body fat as standard: (a) percentage fat by the bioelectrical impedance analysis (BIA) method in women ≥20 years of age; (b) percentage fat by the BIA method in women <20 years of age; (c) percentage fat by the skinfold method in women ≥20 years of age; (d) percentage fat by the skinfold method in women <20 years of age.

The ROC curve analysis indicated that the BMI value that maximised sensitivity and specificity with the skinfold method for body fat calculation among adults was 20·9 kg/m^2^, which had a sensitivity of 86·5 % and specificity of 87·7 %. AUC was 0·95. The analogous value among adolescents was a BMI *Z*-score of –0·33 (which corresponds to approximately a BMI of approximately 19·5 kg/m^2^ at age 14 years and 20·5 kg/m^2^ at age 19 years), which had a sensitivity of 82·4 % and a specificity of 82·2 %. AUC was 0·88 (Table 4 and Fig. 2(c) and (d)). At a BMI of 23 kg/m^2^ (WHO Health Action Point for Asian populations), the sensitivity was low (49·2 %) and specificity was high (98·2 %). Using the 25 kg/m^2^ BMI cut-off (WHO cut-off point, 2004), sensitivity was very low (23 %). Among adolescents, using a BMI *Z*-score of >+1 sd (the WHO recommended cut-point for overweight) produced a sensitivity of 11·7 % and specificity of 99·8 %.

**Table 4.** Summary of receiver operating characteristic curve analysis of BMI/BMI *Z*-score that maximised sensitivity and specificity of body fat classification* among adult and adolescent women

Criterion method	BMI/BMI *Z*-score that maximised sensitivity and specificity	Sensitivity (%)	Specificity (%)	AUC
Women ≥20 years (BMI)				
Percentage fat by skinfold thickness	20·9	86·5	87·7	0·95
Percentage fat by BIA	20·8	83·6	84·2	0·92
Women <20 years (BMI Z-score)				
Percentage fat by skinfold thickness	−0·33	82·4	82·2	0·88
Percentage fat by BIA	−0·36	81·3	80·9	0·87

BIA, bioelectrical impedance analysis.

* Normal = percentage fat ≤30; overweight/excess adiposity = percentage fat >30.

Similar patterns were observed when fat percentage was calculated using BIA. The BMI value that maximised sensitivity and specificity was 20·8 kg/m^2^, resulting in a sensitivity of 83·6 % and specificity of 84·2 % among adults (Table 4). AUC was 0·92. Among adolescents, the BMI *Z*-score that maximised specificity and sensitivity was −0·36, with a sensitivity of 81·3 % and specificity of 80·9 %. AUC was 0·87 (Table 4 and Fig. 2(a) and (b)).

## Discussion

This study builds on the limited work that has examined the relationship between BMI and body fat among South Asian populations, with the goal of evaluating whether existing BMI cut-off points adequately capture excessive body fat in this population. We find that BMI levels corresponding to elevated body fat percentage are remarkably lower in this population than those reported in other Asian and in white European and American populations. Specifically, in our adult population, a BMI of approximately 21 kg/m^2^ maximised the sensitivity and specificity of identifying cases of >30 % body fat, by either skinfold estimates or BIA. This is substantially lower than the WHO Health Action cut-off points (BMI ≥ 23 kg/m^2^) and the WHO recommended standard cut-off point of 25 kg/m^2^. Among adolescents, the analogous value was a BMI *Z*-score of approximately –0·36, which is also substantially lower than the WHO recommended threshold for overweight (≥+1)^(^^24^^)^.

Our findings are consistent with other researchers who have also observed a higher percentage of body fat in Asian Indians at a comparatively low BMI^(^^27^^)^. This is also consistent with recent findings that South Asians have a lower predicted ‘basal BMI’, which is the predicted BMI level for a given population under conditions in which the lack of wealth does not allow for the accumulation of fat mass^(^^16^^,^^28^^)^. Although generally Asian populations tend to have higher body fat at the same level of BMI compared with white American or European populations, it is also widely recognised that there is substantial variation among Asian populations in the level of body fat at the same BMI unit (see Supplementary Tables S1 and S2)^(^^15^^)^. For instance, the WHO Working Group found that, among Asian females, the degree to which BMI was lower for the same level of body fat ranged from less than 0·5 BMI units lower for rural Thai to greater than 3·5 units lower for Hong Kong Chinese. Our study builds on the very small literature examining body fat among South Asians^(^^16^^,^^27^^–^^29^^)^ and improves upon what is known by including a larger sample size and the measurement of body fat by BIA with a validated, population-specific formula for finding percentage body fat. To our knowledge, the only other similar study among South Asians included a small sample of North Indian women (*n* 37), in which Dudeja *et al*.^(^^30^^)^ found that a BMI of 19 kg/m^2^ maximised sensitivity and specificity of classifying body fat as either normal or high (percentage body fat ≥ 30).

With the worldwide increase in overweight and obesity even among populations that have traditionally suffered primarily from undernutrition it is important to examine whether existing BMI cut-off points adequately capture those with excessive adiposity. Our study suggests that the BMI cut-off points that are typically used to proxy increased adiposity and increased health risks are probably too liberal to capture those with moderately excessive body fat in this South Asian population. However, among this thin population, the areas under the ROC curves were high, indicating that the overall performance of BMI for classifying body fat is quite good. Accordingly, BMI may still be an adequate indicator of excessive fatness in this population if lower cut-off points are applied.

Previous literature provides several hypothesised explanations for the high level of adipose tissue seen at lower BMI among Asian populations compared with whites. Visceral adipose tissue tends to be higher in Asian people compared with white Americans or Europeans^(^^31^^)^ and a stocky build tends to be characterised by more bone, muscle mass, connective tissue and less body fat than a more slender build^(^^32^^)^. A study conducted in an Asian population reported that small stature, lower fat-free mass and more slender body size result in more body fat for the same BMI^(^^33^^,^^34^^)^ in Asians compared with whites.

Recent studies suggest that there are large differences in basal BMI among ethnic groups and have estimated that South Asian women have one of the lowest basal BMI levels, which indicates muscoskeletal slenderness^(^^16^^)^. In terms of what makes some populations have more slender builds, physical anthropologists have theorised that climate conditions may drive the slenderness *v*. stockiness of populations^(^^35^^)^. Additionally, recent work has shown that genetic ancestry group explains a substantial proportion of the variance in slenderness between populations^(^^36^^)^. In addition, *in utero* and developmental conditions, dietary composition and physical activity may play roles in determining slender builds^(^^28^^)^.

Strengths of the present study are that body fat percentage was measured by two methods (skinfold and BIA), the two methods were in agreement on mean percentage body fat in the study sample, and that the sample size was large. A limitation of the study is that both the skinfold and the BIA methods for calculating percentage body fat require at least one reference method, though the BIA equation used to calculate fat-free mass was developed against ^2^H_2_O dilution for this population^(^^17^^)^. These women were 3 months postpartum; however, in this sample we have previously shown that by 3 months postpartum, women are back to their pre-pregnancy BMI and body composition status, including reactance and resistance for height^(^^16^^)^. Further research is needed in this population to calculate percentage body fat using reference methods, i.e. underwater weighing, dual-energy X-ray absorptiometry or air displacement plethysmography. Additionally, future research should further examine how non-communicable disease outcomes or intermediate biomarkers are associated with body fat as well as BMI in this population.

In conclusion, traditional BMI cut-offs of 23 and 25 kg/m^2^ had low sensitivity for identifying cases of moderately excessive body fat in rural Bangladeshi women. Our results suggest that BMI can be a diagnostic tool in this population if a substantially lower cut-off of 21 kg/m^2^ is used.

## Supplementary material

For supplementary material accompanying this paper visit http://dx.doi.org/10.1017/jns.2015.32. click here to view supplementary material
