# Supplementary material for: Excessive adiposity at low BMI levels among women in rural Bangladesh
Source: J Nutr Sci. 2016 Feb 17;5:e11. doi: 10.1017/jns.2015.32 (PMC4791523; doi:10.1017/jns.2015.32)
Supplement: Supplementary file 1 [file S2048679015000324sup001.doc]

**Supplementary Table S1.** Comparison of BMI and percentage body fat in Bangladeshi women with Asian and other ethnic groups

| Authors’ name and published year | Country/Ethnic | n | BMI  Mean±SD | P-value^1^ | %Fat  Mmean±SD | P-value^1^ |
| --- | --- | --- | --- | --- | --- | --- |
| Meeuwsen et al, 2010^1^ | UK | 12044 | 25.7±5.1 | <0.0001 | 32.8±8.9 | <0.0001 |
| Sun et al, 2005^2^ | Canada | 491 | 26.0±5.0 | <0.0001 | 34.9±6.7 | <0.0001 |
| Luke et al, 1997^3^ | Black American | 327 | 30.9±7.9 | <0.0001 | 42.2±7.9 | <0.0001 |
| Wang et al, 1994^4^ | Whites | 258 | 23.9±3.4 | <0.0001 | 30.1±8.7 | <0.0001 |
| Gallagher et al, 1996^5^ | Whites | 290 | 23.3±3.7 | <0.0001 | 30.3±8.6 | <0.0001 |
| Gallagher et al, 2000^6^ | White | 225 | 24.5±4.5 | <0.0001 | 32.0 | NA |
| Gallagher et al, 1996^5^ | Blacks | 104 | 27.0±4.3 | <0.0001 | 35.6±8.5 | <0.0001 |
| Gallagher et al, 2000^6^ | Black | 155 | 27.1±4.3 | <0.0001 | 30.7±8.3 | <0.0001 |
| Luke et al, 1997^3^ | Nigerians | 161 | 23.6±3.6 | <0.0001 | 25.1±10.4 | <0.001 |
| Luke et al, 1997^3^ | Jamaicans | 146 | 27.0±6.0 | <0.0001 | 35.3±8.4 | <0.0001 |
| Wang et al, 1994^4^ | Asians | 132 | 22.5±3.3 | <0.0001 | 31.6±6.5 | <0.0001 |
| Gallagher et al, 2000^6^ | Asians | 633 | 23.2±3.9 | <0.0001 | 33.7±7.1 | <0.0001 |
| Yap et al, 2000^7^ | Chinese | 108 | 22.1±4.8 | <0.0001 | 33.3±6.3 | <0.0001 |
| Ko et al, 2001^8^ | Chinese | 3734 | 23.0±3.7 | <0.0001 | 30.1±7.3 | <0.0001 |
| Yap et al, 2000^7^ | Malays | 76 | 24.5±4.8 | <0.0001 | 35.8±6.4 | <0.0001 |
| Dudeja et al, 2001^9^ | Asian Indian | 37 | 23.3±5.5 | <0.0001 | 35.4±5.0 | <0.0001 |
| Yap et al, 2000^7^ | Indians | 107 | 24.9±5.2 | <0.0001 | 35.8±5.6 | <0.0001 |

BMI, body mass index; NA, not available

^1^Statistical significance of difference from the present study.

**Supplementary Table S2.** Comparison of physical measurements among Bangladeshi, East Asian and white American women

| Nutritional predictors | Bangladeshi  n= 1555  mean±SD | Asian^1^  n= 132  mean±SD | White American^1^  n= 258  mean±SD |
| --- | --- | --- | --- |
| Weight, kg | 42.8±5.88 | 54.0±8.0^4^*** | 61.0±9.0^5^*** |
| Height, cm | 149.1±5.25 | 157.0±7.0^4^*** | 163.0±7.0^5^*** |
| BMI, kg/m^2^ | 19.2±2.15 | 22.5±3.3^4^*** | 23.9±3.4^5^*** |
| MUAC (cm) | 22.7±2.11 | 26.9±2.9^4^*** | 27.3±3.2^5^*** |
| UAFA (cm^2^) | 11.4±5.13 | 25.79 | 25.2 |
| Tricep SF (mm) | 10.7±3.98 | 22.0±7.0^4^*** | 21.0±7.0^5^*** |
| Subscapular SF (mm) | 13.8±5.14 | 19.0±8.0^4^*** | 14.0±8.0 |
| Percent body fat^2^ | 23.7±4.81 | 31.6±6.5^4^*** | 30.1±8.7^5^*** |
| Percent body fat^3^ | 23.3±4.88 | NA | NA |

BMI, body mass index; MUAC, mid upper arm circumference; UAFA, upper arm fat area; SF, skinfold thickness; NA, not available

^1^Data from Wang et al, 1994^35^

^2^Fat (%) calculated by 2 DPA systems in Asian and white women^35^; by bioelectrical impedance analysis in Bangladeshi women

^3^Fat (%) calculated by skinfold thickness in Bangladeshi women

^4^Two sample t-test between Bangladeshi women and Asian women

^5^Two sample t-test between Bangladeshi women and White women

***P<0.0001

**References**

(1) [Meeuwsen S](http://www.ncbi.nlm.nih.gov/pubmed?term="Meeuwsen S"%5BAuthor%5D), [Horgan GW](http://www.ncbi.nlm.nih.gov/pubmed?term="Horgan GW"%5BAuthor%5D), [Elia M](http://www.ncbi.nlm.nih.gov/pubmed?term="Elia M"%5BAuthor%5D). The relationship between BMI and percent body fat, measured by bioelectrical impedance, in a large adult sample is curvilinear and influenced by age and sex. [Clin Nutr](http://www.ncbi.nlm.nih.gov/pubmed?term=Meeuwsen S 2010) 2010;29:560-566.

(2) Sun G, French CR, Martin GR, Younghusband B, Green RC, Xie YG et al. [Comparison of multifrequency bioelectrical impedance analysis with dual-energy X-ray absorptiometry for assessment of percentage body fat in a large, healthy population.](http://www.ncbi.nlm.nih.gov/pubmed/15640463) Am J Clin Nutr 2005;81:74-78.

(3) Luke A, Arvizzu DR, Rotimi C, Prewitt E, Forrester T, Wilks R, Ogunbiyi OJ, Schoeller DA, McGee D, Cooper RS. Relation between body mass index and body fat in Black population samples from Nigeria, Jamaica, and the United States. Am J Epidemiol 1997;145:620-628.

(4) Wang J, Thornton JC, Russell M, Burastero S, Heymsfield S, Pierson RN. Asians have lower body mass index (BMI) but higher percent body fat than do whites: comparisons of anthropometric measurements. Am J Clin Nutr 1994;60:23-28.

(5) Gallagher D, Visser M, Sepulveda D, Pierson RN, Harris T, Heymsfield SB. How useful is BMI for comparison of body fatness across age, sex and ethnic groups. Am J Epidemiol 1996;143:228-239.

(6) Gallagher D, Heymsfield SB, Heo M, Jebb SA, Murgatroyd PR, Sakamoto Y. [Healthy percentage body fat ranges: an approach for developing guidelines based on body mass index.](http://www.ncbi.nlm.nih.gov/pubmed/10966886) Am J Clin Nutr 2000;72:694-701.

(7) Yap MD, Schmidt G, van Stavern WA, Deurenberg P. The paradox of low body mass index and high body fat percentage among Chinese, Malays and Indians in Singapore. International Journal of Obesity 2000;24:1011-1017.

(8) [Ko GT](http://www.ncbi.nlm.nih.gov/pubmed?term="Ko GT"%5BAuthor%5D)C, [Tang J](http://www.ncbi.nlm.nih.gov/pubmed?term="Tang J"%5BAuthor%5D), [Chan JC](http://www.ncbi.nlm.nih.gov/pubmed?term="Chan JC"%5BAuthor%5D), [Sung R](http://www.ncbi.nlm.nih.gov/pubmed?term="Sung R"%5BAuthor%5D), [Wu MM](http://www.ncbi.nlm.nih.gov/pubmed?term="Wu MM"%5BAuthor%5D), [Wai HP](http://www.ncbi.nlm.nih.gov/pubmed?term="Wai HP"%5BAuthor%5D), [Chen R](http://www.ncbi.nlm.nih.gov/pubmed?term="Chen R"%5BAuthor%5D). Lower BMI cut-off value to define obesity in Hong Kong Chinese: an analysis based on body fat assessment by bioelectrical impedance. [Br J Nutr](http://www.ncbi.nlm.nih.gov/pubmed/11242492) 2001;85:239-242.

(9) Dudeja V, Misra A, Pandey R, Devina G, Kumar G, Vikram N. BMI does not accurately predict overweight in Asian Indians in northern India. *British Journal of Nutrition* 2001; 86(01):105-112.
